# Undernutrition reduces kisspeptin and neurokinin B expression in castrated male sheep

**DOI:** 10.1530/RAF-20-0025

**Published:** 2020-10-31

**Authors:** Christina M Merkley, Allison N Renwick, Sydney L Shuping, KaLynn Harlow, Jeffrey R Sommer, Casey C Nestor

**Affiliations:** 1 Department of Animal Science, North Carolina State University, Raleigh, North Carolina, USA

**Keywords:** kisspeptin, neurokinin B, sheep, undernutrition, LH

## Abstract

Undernutrition impairs reproductive success through suppression of gonadotropin-releasing hormone (GnRH), and subsequently luteinizing hormone (LH), secretion. Given that kisspeptin and neurokinin B (NKB) neurons in the arcuate nucleus (ARC) of the hypothalamus are thought to play key stimulatory roles in the generation of GnRH/LH pulses, we hypothesized that feed restriction would reduce the ARC mRNA abundance and protein expression of kisspeptin and NKB in young, male sheep. Fourteen wethers (castrated male sheep five months of age) were either fed to maintain (FM; *n* = 6) pre-study body weight or feed-restricted (FR; *n* = 8) to lose 20% of pre-study body weight over 13 weeks. Throughout the study, weekly blood samples were collected and assessed for LH concentration using RIA. At Week 13 of the experiment, animals were killed, heads were perfused with 4% paraformaldehyde, and brain tissue containing the hypothalamus was collected, sectioned, and processed for detection of mRNA (RNAscope) and protein (immunohistochemistry) for kisspeptin and NKB. Mean LH was significantly lower and LH inter-pulse interval was significantly higher in FR wethers compared to FM wethers at the end of the experiment (Week 13). RNAscope analysis revealed significantly fewer cells expressing mRNA for kisspeptin and NKB in FR wethers compared to FM controls, and immunohistochemical analysis revealed significantly fewer immunopositive kisspeptin and NKB cells in FR wethers compared to FM wethers. Taken together, this data supports the idea that long-term feed restriction regulates GnRH/LH secretion through central suppression of kisspeptin and NKB in male sheep.

**Lay summary:**

While undernutrition is known to impair reproduction at the level of the brain, the components responsible for this in the brain remain to be fully understood. Using male sheep we examined the effect of undernutrition on two stimulatory molecules in the brain critical for reproduction: kisspeptin and neurokinin B. Feed restriction for several weeks resulted in decreased luteinizing hormone in the blood indicating reproductive function was suppressed. In addition, undernutrition also reduced both kisspeptin and neurokinin B levels within a region of the brain involved in reproduction, the hypothalamus. Given that they have stimulatory roles in reproduction, we believe that undernutrition acts in the brain to reduce kisspeptin and neurokinin B levels leading to the reduction in luteinizing hormone secretion. In summary, long-term undernutrition inhibits reproductive function in sheep through suppression of kisspeptin and neurokinin B within the brain.

## Introduction

The capacity for reproduction first occurs at puberty and is the result of an activated hypothalamic–pituitary–gonadal axis. As a critical window of time in physiological development, puberty onset is heralded at the neuroendocrine level by an elevation of pulsatile gonadotropin-releasing hormone (GnRH) secretion from the CNS, which in turn elicits an increase of luteinizing hormone (LH) secretion from the anterior pituitary. It has been known for decades that undernutrition delays puberty onset in mammals (Glass & Swerdloff 1980, Chakravarty *et al.* 1982, Fitzgerald *et al.* 1982, Foster & Olster 1985, Day *et al.* 1986) and with evidence that insufficient energy intake (caloric restriction) reduces GnRH release into the hypophyseal portal circulation (I’Anson *et al.* 2000), there is a central mechanism whereby undernutrition impairs reproduction through a reduction in GnRH/LH secretion. Given that direct nutritional regulation of GnRH neurons is unlikely as they are devoid of leptin and insulin receptors (Finn *et al.* 1998, Hakansson *et al.* 1998, Quennell *et al.* 2009, Louis *et al.* 2011, Cernea *et al.* 2016), the central mechanism whereby undernutrition regulates GnRH/LH secretion is likely mediated by afferent input to GnRH neurons.

Mutations in the genes encoding the neuropeptide kisspeptin or its receptor, Kiss1R (also known as G-protein coupled receptor 54, GPR54) result in impairment of pubertal maturation and reproductive function in humans and mice (de Roux *et al.* 2003, Seminara *et al.* 2003, Topaloglu *et al.* 2012), which is compelling evidence that kisspeptin is an essential component of normal pubertal development and fertility. With nearly all GnRH neurons expressing Kiss1R (Irwig *et al.* 2004, Herbison *et al.* 2010, Smith *et al.* 2011, Bosch *et al.* 2013) and the robust stimulatory actions of kisspeptin on GnRH and gonadotropin release in numerous mammalian species (Irwig *et al.* 2004, Navarro *et al.* 2004, Shahab *et al.* 2005, Mason *et al.* 2007, Lents *et al.* 2008, Jayasena *et al.* 2009, Magee *et al.* 2009, Ohkura *et al.* 2009), kisspeptin is believed to provide the direct stimulatory drive to GnRH neurons. Similar to kisspeptin, neurokinin B (NKB) is also essential for puberty, as loss-of-function mutations in the genes encoding NKB or its receptor, neurokinin 3 receptor (NK3R), result in failure of pubertal progression and infertility in humans (Topaloglu *et al.* 2009). Furthermore, administration of NKB or senktide (an NK3R receptor agonist) has been shown to stimulate LH secretion in multiple species (Billings *et al.* 2010, Ramaswamy *et al.* 2010, Wakabayashi *et al.* 2010, Navarro *et al.* 2011, Nestor *et al.* 2012). Since kisspeptin neurons in the arcuate nucleus (ARC) of the hypothalamus highly express NK3R, while GnRH neurons appear to be devoid of NK3R (Amstalden *et al.* 2010, Ahn *et al.* 2015), NKB action is thought to occur through stimulation of ARC kisspeptin which in turn stimulates GnRH neurons. In support of this, work in rodents has shown that removal of Kiss1R signaling abrogates the response to senktide (Garcia-Galiano *et al.* 2012, Grachev *et al.* 2012*b*) and thus demonstrates that NKB signaling is upstream of kisspeptin signaling.

Given their dominant stimulatory roles in reproduction, kisspeptin and NKB may also play an important part in mediating the effect of undernutrition on GnRH/LH secretion. Unlike GnRH neurons, ARC kisspeptin appear to express receptors for both leptin (Smith *et al.* 2006, Backholer *et al.* 2010*b*, Cravo *et al.* 2011) and insulin (Cernea *et al.* 2016), and thus may be a direct target of these key metabolic hormones. In addition, food deprivation (total food withdrawal for 48–72 h) has been shown to reduce hypothalamic mRNA abundance of kisspeptin (Castellano *et al.* 2005, True *et al.* 2011, Navarro *et al.* 2012) and protein expression of kisspeptin in the ARC (Polkowska *et al.* 2015). Furthermore, while short-term feed restriction (30% feed withdrawal for 10 days) in pigs elicit no change in mRNA for kisspeptin and increases mRNA for NKB (Thorson *et al.* 2018), others have shown in mice, rats, and sheep that long-term food restriction (30–60% food withdrawal for several weeks/months) is capable of reducing ARC mRNA abundance of kisspeptin (Backholer *et al.* 2010*a*, True *et al.* 2011, Yang *et al.* 2016) and NKB (True *et al.* 2011, Yang *et al.* 2016). While this evidence provides valuable insight into the central link between energy balance and reproduction, data on the effects of undernutrition on kisspeptin and NKB in males is limited, and thus far has only been reported for food deprivation in mice (Castellano *et al.* 2005). Moreover, previous reports have focused on how undernutrition impacts either mRNA or protein but have yet to report both message and protein within an experiment. Therefore, in the present study, we used a chronic feed restriction model to examine the effects of undernutrition on mRNA and protein for kisspeptin and NKB in the ARC of young, castrated male sheep (wethers). The use of wethers in these experiments enabled us to examine direct nutritional effects on LH secretion independent of changes in sensitivity to gonadal steroid feedback that may result from undernutrition. We hypothesized that chronic feed restriction, which would suppress LH secretion, would reduce both mRNA abundance and protein expression of kisspeptin and NKB. To test this hypothesis, we used a relatively new fluorescent *in situ* hybridization technique, RNAscope, to assess mRNA and classic immunohistochemistry for protein detection.

## Materials and methods

### Animals

Fourteen Suffolk wethers (male sheep castrated between four and six weeks of age) were approximately five months of age at the start of the study which was conducted from July through October. Prior to the study, wethers were housed in an open barn for a minimum of 14 days, and received open access to water and hay supplemented with the experimental diet (crude protein 12%, crude fat 2.5%, crude fiber 5.0%; Mule City Specialty Feeds, Benson, NC). Once moved indoors for the duration of the study, all sheep were housed individually, provided with water ad libitum and fed once daily with the experimental diet. Indoor lighting simulated changes in natural day length throughout the study. Blood samples were obtained using jugular venipuncture, collected into heparinized tubes, and plasma was stored at −20°C. All procedures were approved by the North Carolina State University Animal Care and Use Committee and followed the National Institutes of Health guidelines for use of animals in research.

### Experimental design

**Figure 1:**
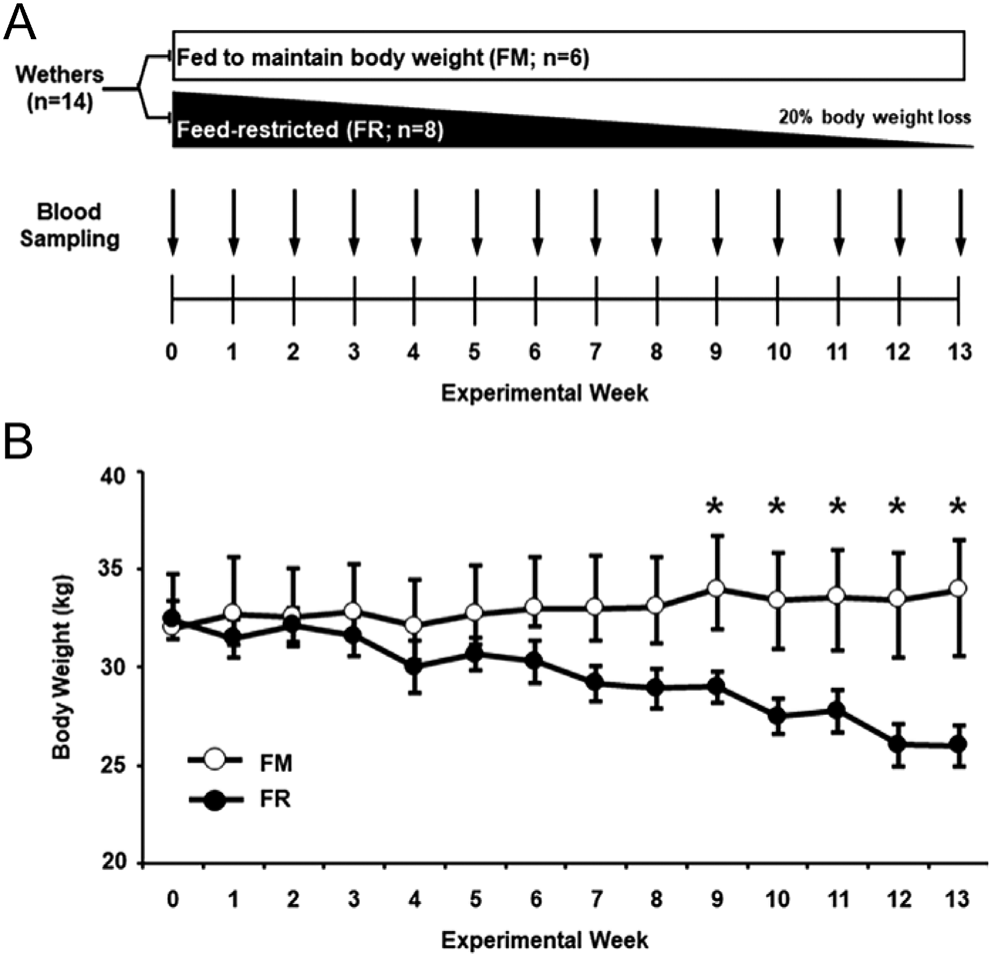
(A) At Week 0, wethers were divided into fed to maintain body weight (FM; *n* = 6) and feed-restricted to lose body weight (FR; *n,*= 8) groups. Weekly blood sampling was conducted from the start of the experiment (Week 0) through the end of the study (Week 13). (B) From Week 9 to Week 13, average body weights (mean ± s.e.m.) were significantly lower in FR wethers compared to FM wethers (*P < 0.05).

The experimental design for this study is depicted in Fig. 1A. Briefly, 14 wethers were divided into one of two groups: fed to maintain body weight (FM; *n* = 6) or feed-restricted to lose body weight (FR; *n* = 8). Based on previous reports in sheep (Foster & Olster 1985, Adam *et al.* 1997, McManus *et al.* 2005), each animal in the FM group was fed the experimental diet in order to maintain their pre-study body weight, while each animal in the FR group was fed less of the same diet in order to lose 20% of their own pre-study body weight over the course of 13 weeks. Body weight data were collected weekly throughout the experiment, and feed intake for each group was adjusted to produce the desired change in weight. Peripheral blood samples were collected weekly (from Weeks 0 to 13) via jugular venipuncture every 12 min for 4.5 h.

### Tissue collection

Tissue was collected as previously described (Foradori *et al.* 2006). Briefly, at the end of the experiment (Week 13), all wethers were heparinized (20,000 U, intravenous) and killed with an intravenous overdose of sodium pentobarbital (Euthasol; Patterson Veterinary, Greeley, CO). Heads were removed and perfused via the carotid arteries with four liters of 4% paraformaldehyde (PFA) in 0.1 M PBS (pH 7.4) containing 0.1% sodium nitrite. Blocks of tissue containing the hypothalamus were removed and stored in 4% PFA for 24 h at 4°C and transferred to a 20% sucrose solution until sectioning. Frozen coronal sections were cut at 50 µm with a freezing microtome into five parallel series and stored in cryopreservative solution until used for RNAscope and immunohistochemistry.

### RNA scope *in situ* hybridization

For the detection of mRNA for kisspeptin and NKB, RNAscope was performed on PFA-fixed hemisections from FM and FR wethers mounted on Superfrost/Plus microscope slides (Fisher Scientific). Three sections in the middle ARC were selected in each animal from a series of every fifth hypothalamic section (250 µm apart), with the middle ARC defined as the level of the tubero-infundibular sulcus until the beginning of the formation of the mammillary recess of the third ventricle (Merkley *et al.* 2012, Weems *et al.* 2016). Each full coronal section was cut at midline with only one side of the tissue section used for RNAscope. *In situ* hybridization was performed based on instructions from Advanced Cell Diagnostics and technical recommendations with minor modifications using the RNAscope Multiplex Fluorescent Reagent Kit v2 (Advanced Cell Diagnostics, Newark, CA; cat# 323100). All incubations between 40 and 60°C were conducted using an ACD HybEZ II Hybridization System with EZ-Batch Slide System (Advanced Cell Diagnostics; cat# 321710). On day 1, hemisections were washed overnight in 0.1 M PBS at 4°C on a rocking shaker to remove excess cryoprotectant. On day 2, hemisections were mounted onto microscope slides and allowed to dry for 2 h. Then slides with sections were heated at 60°C for 90 min, submerged in chilled 4% PFA at 4°C for 1 h, and rinsed four times in 0.1 M PBS (5 min/rinse). Slides were then incubated in increasing concentrations of ethanol (50, 70, 100, and 100%) for 5 min at each concentration. Slides were then allowed to dry at room temperature (RT) for 5 min and then incubated in Hydrogen Peroxide solution (10 min at RT; Advanced Cell Diagnostics, cat# 322335). Next, slides were briefly rinsed with deionized water five times, treated with Target Retrieval solution (15 min at 100°C; Advanced Cell Diagnostics, cat# 322001), then rinsed in deionized water five times followed by quick submersion in 100% ethanol five times and allowed to air dry. A hydrophobic barrier was then created around the tissue using an ImmEdge Pen (Advanced Cell Diagnostics; cat# 310018), and slides were stored overnight at 4°C. On day 3, sections were treated with RNAscope® Protease III (30 min at 40°C; Advanced Cell Diagnostics, cat# 322337), and subsequently incubated with RNAscope target (kisspeptin, Oa-KISS1-C3, cat# 497471-C3; NKB, Oa-TAC3-O1, cat# 481411) and control probes (positive controls, Oa-UBC-C3, cat#516181-C3 and Oa-POLR2A, cat# 516171; negative control, 3-plex Negative Control Probe, cat# 320871) for 2 h at 40°C. Next, slides were washed twice with 1x Wash Buffer (Advanced Cell Diagnostics, cat# 310091; 2 min/rinse at RT) followed by sequential tissue application of 50 µl of the following each for 30 min at 40°C with 2 min washes using 1x Wash Buffer between applications: RNAscope Multiplex FL v2 Amp 1 (Advanced Cell Diagnostics, cat# 323101), RNAscope Multiplex FL v2 Amp 2 (Advanced Cell Diagnostics, cat# 323102), and RNAscope Multiplex FL v2 Amp 3 (Advanced Cell Diagnostics, cat# 323103). Following final incubation with Amp 3, slides were rinsed with 1x Wash Buffer twice (2 min/rinse at RT) followed by application of RNAscope Multiplex FL v2 HRP C1 (15 min at 40°C; Advanced Cell Diagnostics, cat#323104). Next, sections were incubated with 150 µL per slide of Opal 570 (Fisher Scientific; cat# NC1601878) in RNAscope TSA buffer (Advanced Cell Diagnostics, cat# 322809) at a final concentration of 1:1500 for 30 min at 40°C. Following a rinse with 1 x Wash Buffer twice (2 min/rinse at RT), 50 µL of RNAscope® Multiplex FL v2 HRP Blocker (Advanced Cell Diagnostics, cat# 323107) was applied to tissue for 15 min at 40°C. Slides were then rinsed with 1x Wash Buffer twice (2 min/rinse at RT) followed by application of RNAscope Multiplex FL v2 HRP-C3 (15 min at 40°C; Advanced Cell Diagnostics, cat# 323106). Sections were next incubated with 150 µL per slide of Opal 690 (Fisher Scientific; cat# NC1605064) in RNAscope TSA buffer (Advanced Cell Diagnostics, cat# 322809) at a final concentration of 1:1500 for 30 min at 40°C, and followed by rinsing of the slide in 1x Wash Buffer twice (2 min/rinse at RT). Then, 50 µL of RNAscope® Multiplex FL v2 HRP Blocker (Advanced Cell Diagnostics, cat# 323107) was applied to tissue for 15 min at 40°C. Finally, slides were coverslipped with ProLong Gold Antifade Mountant (Fisher Scientific, cat# P36930) and stored at 4°C until image acquisition.

### Immunohistochemistry for kisspeptin or NKB

For the detection of each antigen, immunohistochemical procedures were performed on free-floating hemisections from FM and FR wethers. Four sections in the middle ARC were selected in each animal from a series of every fifth hypothalamic section (as described above for RNAscope). Each full coronal section was cut at midline with the left and right side of the brain tissue section used for single-label immunoperoxidase detection of kisspeptin and NKB, respectively. See Table 1 for information on all primary antibodies used herein. On day 1, sections were washed overnight in 0.1 M PB at 4°C on a rocking shaker to remove excess cryoprotectant. All subsequent steps were conducted at RT (except where specified). On day 2, sections were washed four times (5 min each) in 0.1 M PBS (pH 7.4), then placed into 10% H_2_O_2_ (diluted in 0.1 M PBS) for 10 min followed by four washes (5 min each) in PBS. Tissue was then incubated for 1 h in a PBS solution containing 0.4% Triton-X (Sigma Aldrich) and 20% normal goat serum (NGS; Jackson ImmunoResearch Laboratories, Inc., West Grove, PA) for kisspeptin or 4% NGS (Jackson) for NKB. Next, to identify kisspeptin or NKB, hemisections were incubated in either primary antibody rabbit anti-kisspeptin-10 (1:50,000; Gift from I. Franceschini, #566) or rabbit anti-NKB (1:8000; Phoenix Pharmaceuticals, Inc., Burlingame, CA, cat# H-046-26), diluted in PBS containing 0.4% Triton X-100 and 4% NGS for 17 h. On day 3, biotinylated goat anti-rabbit IgG (1:500; Jackson) and Vectastain ABC-elite (1:500; Vector Laboratories, Burlingame, CA) were applied sequentially for 1 h each with four washes (5 min each) of PBS between incubations. Hemisections were then placed in a 3,3’-diaminobenzidine tetrahydrochloride (DAB; Fisher Scientific, cat# AC328005000) solution (10 mg DAB in 50 mL of PB with 20 µL of 30% H_2_O_2_ added just prior to use) for 10 min. After four washes (5 min each) in PB, sections were mounted on Superfrost/Plus microscope slides (Fisher Scientific), air dried, dehydrated using a series of increasing alcohol baths, and coverslipped using DPX Mounting Medium (Sigma Aldrich, cat# 06522).

**Table 1:** Primary antibody information for immunohistochemistry.

Protein	Antigen sequence	Species raised	Manufacturer. Catalog #	References
Kisspeptin-10	YNWNSFGLRY-NH2 Residues 43-52 of mouse metastin	Rabbit; polyclonal	Gift from I. Franceschini, #566	Franceschini *et al.* (2006), Goodman *et al.* (2007), Smith *et al.* (2011), De Bond *et al.* (2013)
Neurokinin B	DMHDFFVGLM-NH2	Rabbit; polyclonal	Phoenix, H-046-26	Weems *et al.* (2016)

### Primary antibody validation for immunohistochemistry

The antibody used to detect kisspeptin in this study has been previously validated for use in sheep neural tissue (Franceschini *et al.* 2006). To validate the specificity of the rabbit anti-NKB antibody (Phoenix) in sheep neural tissue, we conducted a pre-absorption experiment in which the rabbit anti-NKB (1:8000; Phoenix) was incubated with increasing concentrations of the NKB peptide (0, 10, 30 and 75 µg/mL; Phoenix, cat # 046-26) overnight at RT. These working solutions were incubated with two ARC hemisections per animal from four wethers (*n* = 2/group) using the single- label immunoperoxidase detection protocol for NKB described above. Pre-absorption of the rabbit anti-NKB with peptide concentrations 10, 30, and 75 µg/mL abolished all NKB-immunoreactivity in the ovine ARC, while the positive control (anti-NKB + 0 µg/mL peptide) showed NKB immunopositive cells and fibers (data not shown).

### Data analysis

#### LH assay

LH concentrations were measured in duplicate with an RIA using 50–100 µL of plasma and reagents purchased from the National Hormone and Peptide Program (Torrance, CA) as previously described (Whisnant & Goodman 1988). Analysis of LH data included mean LH concentration, LH inter-pulse interval, and LH pulse amplitude. Individual pulses of LH were identified using previously described criteria (Goodman & Karsch 1980). Briefly, there were three main criteria: (1) the peak must exceed the sensitivity of the assay, (2) a peak must occur within two data points of the previous nadir, and (3) the peak must exceed a 95% CI of the previous and following nadirs. LH assay sensitivity was 0.20 ng/mL with intra- and inter-assay coefficients of variation being 5.69 and 13.89%, respectively.

### RNAscope *in situ* hybridization

Hemisections processed for simultaneous detection of mRNA for kisspeptin and NKB were analyzed within 3 weeks following the RNAscope procedure, using a Zeiss 880 confocal microscope. The number of cells expressing kisspeptin or NKB mRNA were quantified in the middle ARC by a blinded observer, using images taken with a Plan Apochromat 10x/0.45 objective, with consistent acquisition settings for all hemisections. Images of each hemisection were uploaded to Adobe Photoshop (Adobe, Inc., San Jose, CA), where individual cells were marked in a superimposed image layer. Then, Image J was used to quantify the number of marked cells within the region of interest. Given the diffuse labeling of both kisspeptin and NKB, a product of abundant mRNA expression (Jolly *et al.* 2019) (personal communication with Advanced Cell Diagnostics Technical Support), individual cells that expressed mRNA for kisspeptin and NKB (24–30 cells/animal) were readily identified and randomly selected for integrated density analysis. Confocal z-stack images that encompassed each cell were captured at 1 µm optical sections with a Plan Apochromat 63x/1.4 oil objective and acquisition settings were identical for all images including positive and negative control probes. Following the acquisition, an experimenter blinded to the treatment group opened optical slice images in Image J (NIH), converted images to 16-bit, and applied a region of interest (312 pixels x 312 pixels) directly over each cell to determine integrated density. An auto-threshold was recorded for each channel corresponding to the specific label in all optical slices. An average of these thresholds was determined for each of the channels and used as the fixed threshold intensity for the integrated density analysis. Three optical slices from the center of each cell as determined by the extent of detectable signal throughout the cell were used for analysis, with the sum of the integrated density values calculated per cell and then averaged per animal for statistical comparison.

### Immunohistochemistry

Single-labeled kisspeptin and NKB cells were quantified in the middle ARC by two independent, blinded observers, using brightfield images captured using a Laxco SeBa 2 Digital Microscope System (Fisher Scientific, cat# NC1347978) with a Plan Apochromat 10x/0.25 objective and a Plan Apochromat 20x/0.40 objective with consistent camera settings across all hemisections. Kisspeptin-immunoreactive (ir) and NKB-ir cell bodies were assessed by an experimenter blinded to treatment groups and identified by brown cytoplasmic staining, and those with defined borders were included in the analysis (Merkley *et al.* 2012, Nestor *et al.* 2012). For all animals, images of each hemisection were uploaded to Adobe Photoshop (Adobe, Inc., San Jose, CA), where individual cells were marked in a superimposed image layer. Then, Image J was used to quantify the number of marked cells within the region of interest.

### Statistical analysis

Average body weights, mean LH concentrations, LH inter-pulse interval, and LH pulse amplitude were analyzed using a repeated measures two-way ANOVA. Data for RNAscope *in situ* hybridization and immunohistochemistry were analyzed using an unpaired, Student’s *t*-test. Differences were considered to be statistically significant at *P* < 0.05. All analyses were performed using Sigma Plot 11.0 (San Jose, CA).

## Results

### Body weights

Average weekly body weights from FM wethers and FR wethers throughout the study are illustrated in Fig. 1B. There was a treatment x time interaction (*P* < 0.001) for body weight between groups. At the beginning of the experiment (Week 0), average body weights were not significantly different between FM and FR groups; however, starting at Week 9 through the end of the study (Week 13), average body weights were significantly lower (*P* < 0.05) in FR wethers compared to FM wethers. As designed, the average percent change in pre-study body weight for the FM and FR wethers at Week 13 was 6.79 + 3.4 and -19.82 ± 1.6%, respectively.

### LH data

**Figure 2:**
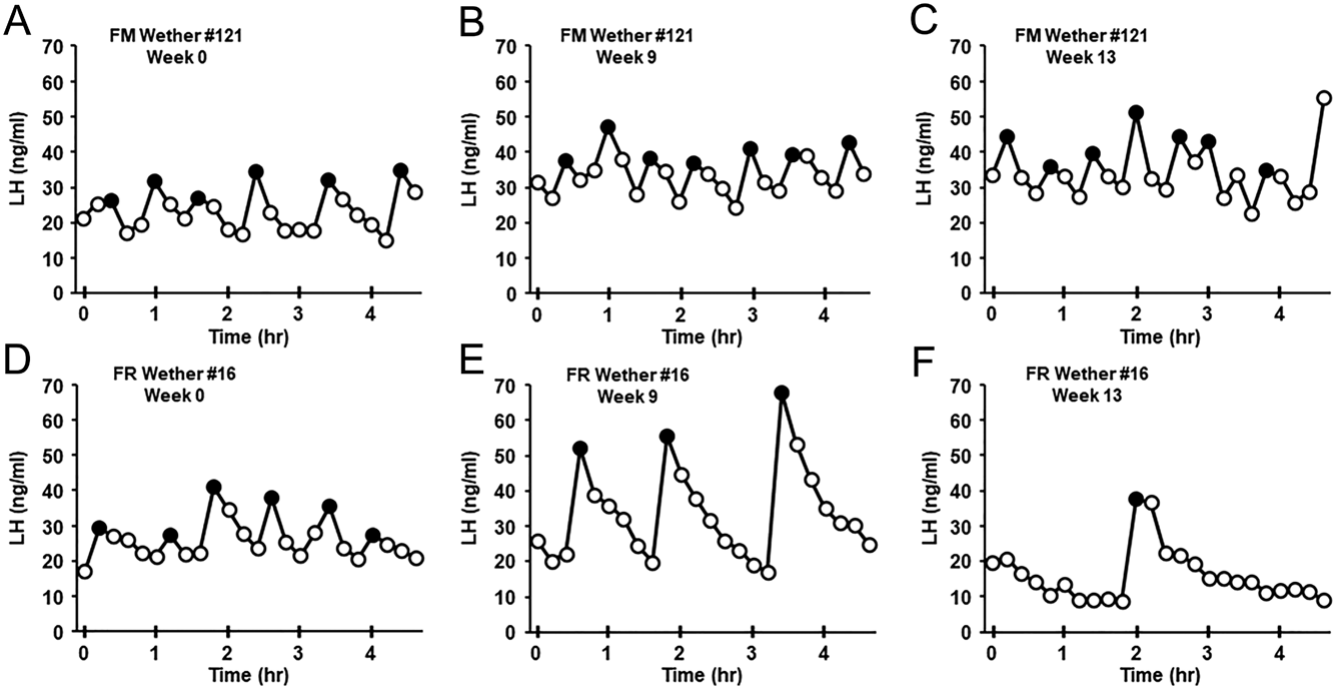
Representative LH profiles (mean ± s.e.m.) from a fed to maintain (FM) wether (A, B and C) and a feed-restricted (FR) wether (D, E and F) on Week 0 (A, D), Week 9 (B, E), and Week 13 (C, F). LH pulses are denoted by closed circles.

**Figure 3:**
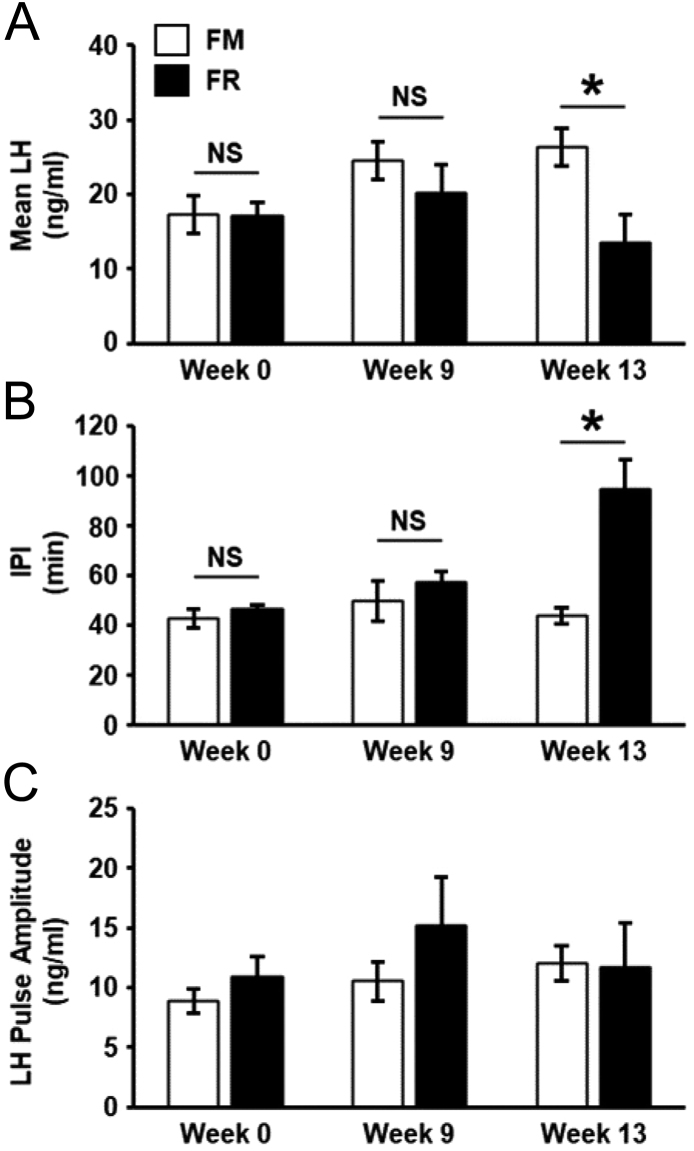
Mean (±s.e.m.) concentrations of LH (A), inter-pulse interval (IPI; B) and LH pulse amplitude (C) from fed to maintain (FM; *n* = 6) and feed-restricted (FR; *n* = 8) wethers. (A) Mean LH was significantly lower in FR animals compared to FM wethers at Week 13 (*P < 0.05), while a significant difference was not detected at Week 0 or 9. (B) IPI was significantly greater in FR wethers compared to FM wethers at Week 13 (*P < 0.05), but not significantly different at Weeks 0 or 9. (C) There was no significant effect of feed restriction on LH pulse amplitude (*P* > 0.05).

Representative LH pulse profiles from FM and FR wethers are shown in Fig. 2. Quantification of mean LH concentration, LH inter-pulse interval, and LH pulse amplitude for FM and FR wethers at Weeks 0, 9, and 13 are illustrated in Fig. 3. There was a significant interaction (*P* < 0.001) of time x treatment for mean LH concentration and LH inter-pulse interval. While not significantly different at Weeks 0 and 9, mean LH concentration at Week 13 was significantly lower (*P* < 0.05) in FR wethers compared to FM wethers (Fig. 3A). In addition, LH inter-pulse interval was significantly greater in FR wethers compared to FM wethers at Week 13 (*P* < 0.05; Fig. 3B), but at Weeks 0 and 9 did not show a significant difference between groups. No significant differences between groups were detected for LH pulse amplitude (Fig. 3C).

### Kisspeptin data

**Figure 4:**
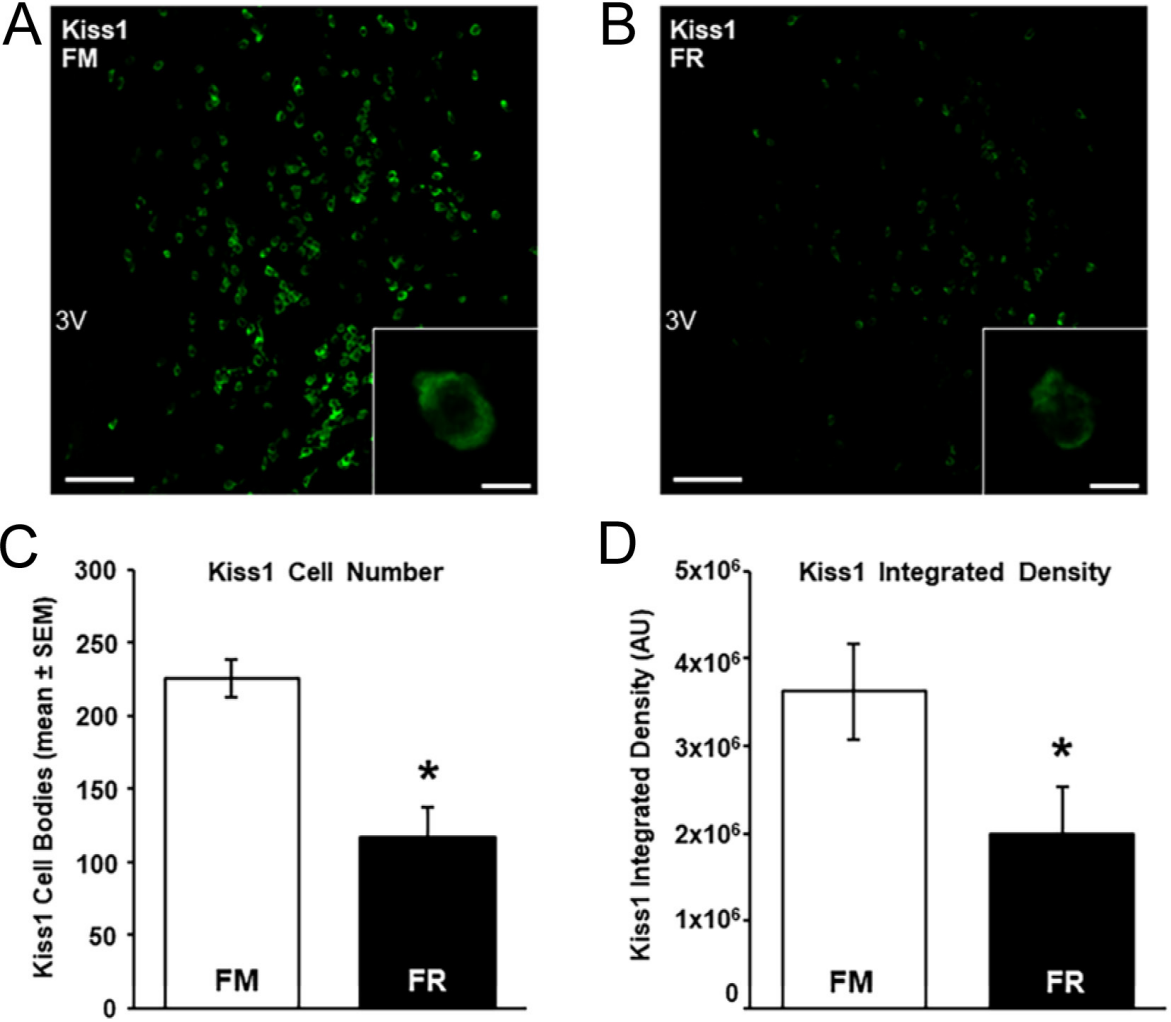
Kiss1 mRNA in the arcuate nucleus (ARC) of fed to maintain (FM) and feed-restricted (FR) wethers. Confocal images (using a 10x objective) of Kiss1 cells from an FM (A) and FR (B) wether. Insets show high magnification confocal images (1 µm optical section, 63x objective) of representative Kiss1 cells in a FM (A inset) and a FR (B inset) wether. (C) Mean (±s.e.m.) number of Kiss1 cell bodies in the ARC were significantly lower (*P < 0.05) in FR wethers compared to FM wethers. (D) Mean (±s.e.m.) integrated density of Kiss1 mRNA per ARC cell was significantly lower (*P < 0.05) in FR wethers (*n* = 8) compared to FM wethers (*n* = 6). Scale bars, 100 µm. Scale bars for insets, 10 µm. For print clarity, Kiss1 mRNA has been pseudocolored green in these images. 3V, third cerebroventricle.

We assessed changes in kisspeptin mRNA and protein in the ARC between FM and FR wethers using RNAscope and immunohistochemistry, respectively. Cells expressing mRNA for kisspeptin were readily visible in the ARC of both FM wethers (Fig. 4A) and FR wethers (Fig. 4B). The average number of kisspeptin cells (Fig. 4C) and the expression of mRNA transcript for kisspeptin per cell (Fig. 4D) were significantly lower (*P* < 0.05) in FR wethers compared to FM wethers.

**Figure 5:**
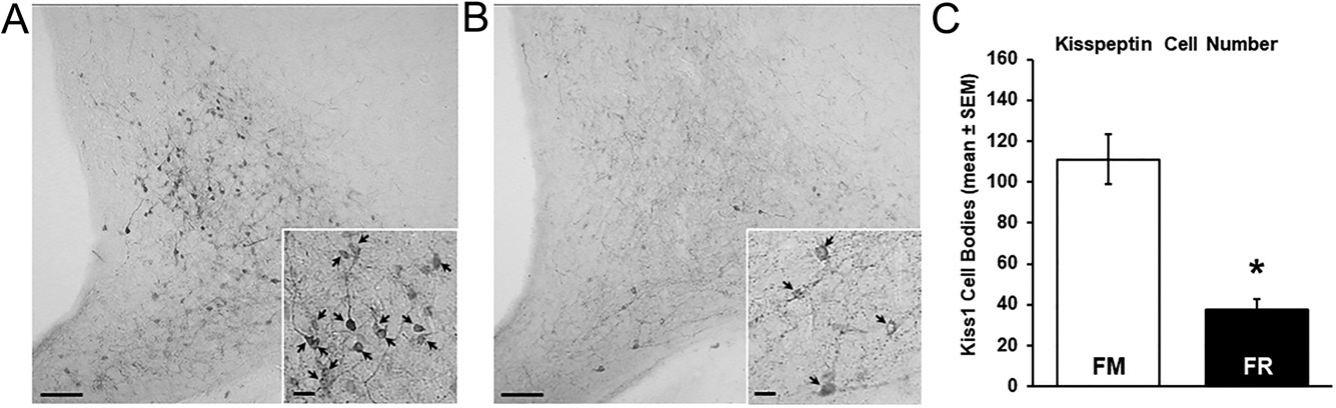
(A and B) Representative photomicrographs (using a 10x objective) showing kisspeptin-immunoreactive (ir) cells and fibers in the middle arcuate nucleus (ARC) from a fed to maintain (FM; A) wether and a feed-restricted (FR; B) wether. Insets (using a 20x objective), arrows indicate kisspeptin-ir cell bodies. (C) Mean (±s.e.m.) number of kisspeptin-ir cell bodies per hemisection in the ARC was significantly lower (*P < 0.05) in FR wethers (*n* = 8) compared to FM wethers (*n* = 6). Scale bars, 200 µm. Scale bars for insets, 25 µm.

Cell bodies and fibers expressing protein for kisspeptin were readily visible in the ARC of both FM (Fig. 5A) and FR wethers (Fig. 5B). Analysis of kisspeptin-immunopositive perikarya revealed that FR wethers had significantly fewer numbers of kisspeptin cell bodies (*P* < 0.01) compared to FM wethers (Fig. 5C).

### NKB data

**Figure 6:**
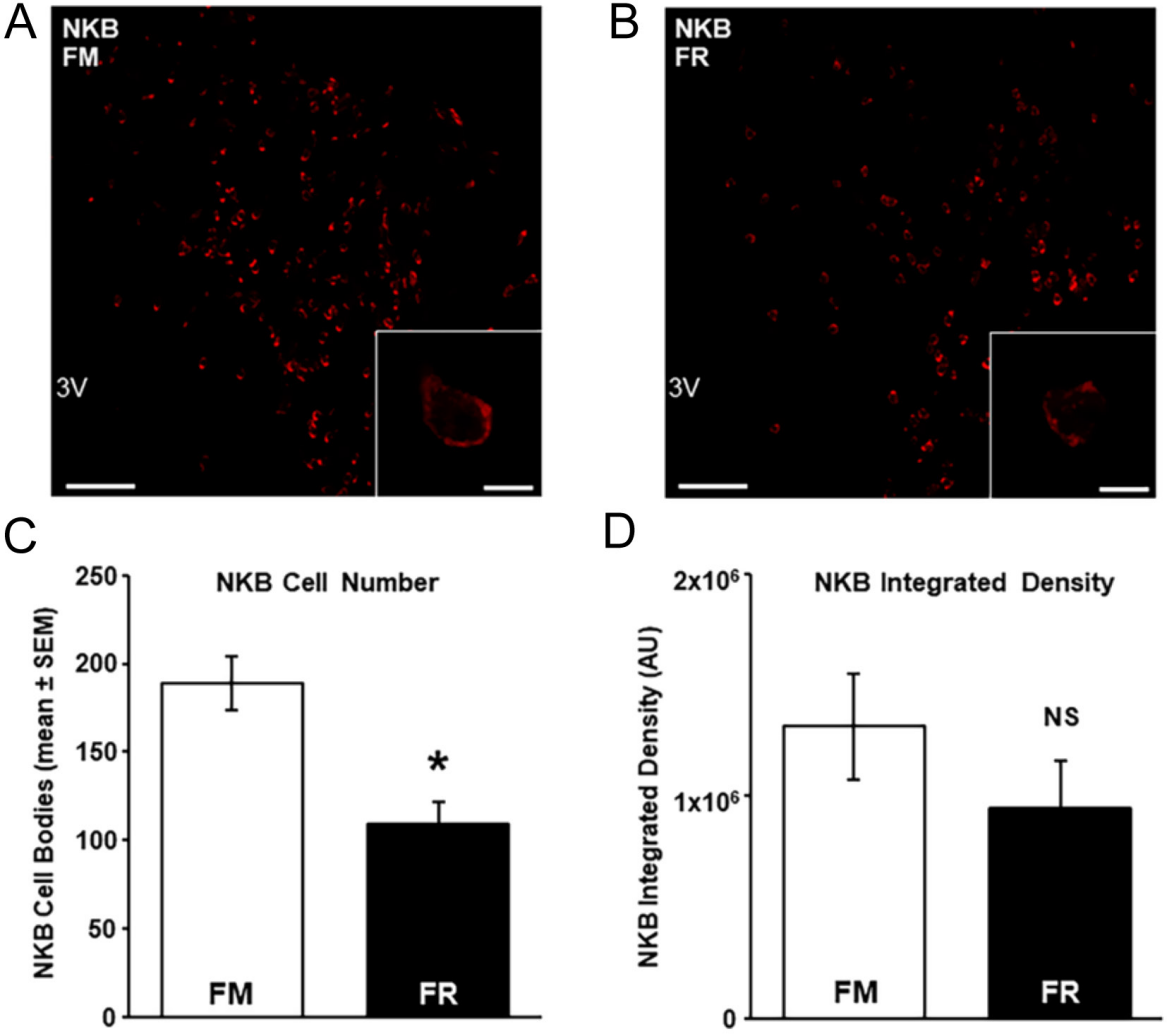
NKB mRNA in the arcuate nucleus (ARC) of fed to maintain (FM) and feed-restricted (FR) wethers. Confocal images (using a 10x objective)) of NKB cells from an FM (A) and FR wether (B). Insets show high magnification confocal images (1 µm optical section, 63x objective) of representative NKB cells in a FM (A inset) and a FR (B inset) wether. (C) Mean (±s.e.m.) number of NKB cell bodies in the ARC were significantly fewer (*P < 0.05) in FR wethers (*n* = 8) compared to FM wethers (*n* = 6). (D) Mean integrated density of NKB mRNA per ARC cell was not significantly (NS) different between groups. Scale bars, 100 µm. Scale bars for insets, 10 µm. 3V, third cerebroventricle.

In the same cells that expressed mRNA for kisspeptin, we assessed changes in NKB mRNA and protein in the ARC between FM and FR wethers. Cells expressing mRNA for NKB were readily detectable in the ARC of both FM wethers (Fig. 6A) and FR wethers (Fig. 6B). The average number of NKB cells was significantly lower (*P* < 0.05) in FR wethers compared to FM wethers (Fig. 6C), but the expression of mRNA transcript per cell did not differ between groups (*P* > 0.05; Fig. 6D). The percentage of NKB cells that expressed mRNA for kisspeptin in FM and FR wethers was 60.5 ± 6 and 64.2 ± 5%, respectively, while the percentage of kisspeptin cells that expressed mRNA for NKB in FM and FR wethers was 52.0 ± 7 and 65.7 ± 5%, respectively.

**Figure 7:**
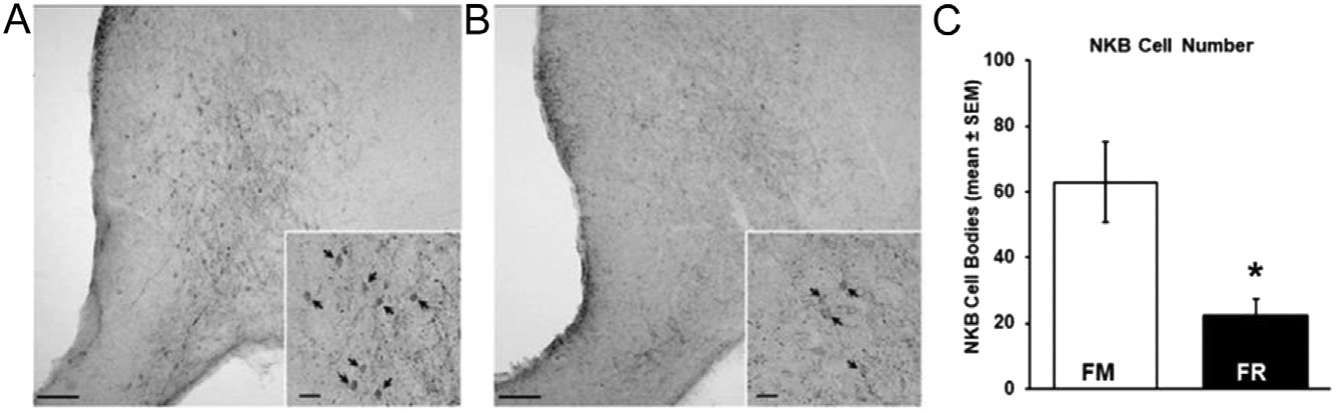
(A and B) Representative photomicrographs (using a 10x objective) showing NKB-immunoreactive (ir) cells and fibers in the middle arcuate nucleus (ARC) from a fed to maintain (FM; A) wether and a feed-restricted (FR; B) wether. Insets (using a 20x objective), arrows indicate NKB-ir cell bodies. (C) Mean (±s.e.m.) number of NKB-ir cells per hemisection in the middle ARC was significantly lower (*P < 0.05) in FR wethers (*n* = 8) compared to FM wethers (*n* = 6). Scale bars, 200 µm. Scale bars for insets, 25 µm.

Within the ARC of both FM (Fig. 7A) and FR wethers (Fig. 7B), cell bodies and fibers expressing protein for NKB were readily visible. Analysis of the number of NKB-ir cells revealed that FR wethers had significantly fewer (*P* < 0.01) NKB cell bodies compared to FM wethers (Fig. 7C).

## Discussion

The data herein provide evidence of a role for kisspeptin and NKB in the central regulation of LH secretion during undernutrition in male sheep. Chronic feed-restriction in wethers, which produced a significant reduction in LH secretion, resulted in fewer ARC neurons expressing mRNA and protein for kisspeptin and reduced mRNA abundance of kisspeptin in the remaining kisspeptin neurons. In addition, this model of undernutrition resulted in fewer ARC neurons expressing mRNA and protein for NKB. Although there was not a significant effect of feed-restriction on mRNA abundance of NKB in the remaining NKB neurons, the overall nutritionally-induced inhibition of kisspeptin and NKB provides important neuroanatomical evidence for the central regulation of these neurons during undernutrition.

Sufficient energy intake is an important component of proper reproductive function and as such, it is generally accepted that undernutrition has the capacity to impair reproduction. Earlier work in sheep has demonstrated that undernutrition reduces GnRH release from the hypothalamus (Ebling *et al.* 1990, Prasad *et al.* 1993, I’Anson *et al.* 2000) lending support to the idea that a negative energy balance is sensed at the level of the brain resulting in lower GnRH, and subsequently LH, secretion. Based on previous models in young, gonadectomized sheep (Foster & Olster 1985, Adam *et al.* 1997, McManus *et al.* 2005), we established a model of undernutrition in wethers that produces a robust reduction of LH secretion. Given that our study used LH secretion as an index of GnRH release, it is possible that undernutrition decreased pituitary responsiveness to GnRH. However, since others have shown that exogenous GnRH administration elicits LH secretion in growth-restricted sheep (Ebling *et al.* 1990) and rodents (Bronson 1988), it is more likely that the primary mechanism whereby undernutrition decreases LH secretion is through a central-mediated pathway regulating GnRH release, given data herein that feed restriction reduced LH pulse frequency, but not LH pulse amplitude. Moreover, as the final common conduit from the CNS controlling reproduction, GnRH neurons play an integral part of the central mechanism whereby undernutrition limits reproduction, but GnRH neurons do not appear to be direct targets of leptin or insulin (Finn *et al.* 1998, Quennell *et al.* 2009, Louis *et al.* 2011, Cernea *et al.* 2016). Therefore, afferent neurons likely mediate the loss of these satiety signals during times of undernutrition.

As a potent stimulator of GnRH neurons, kisspeptin plays an essential role in pulsatile GnRH/LH secretion and is one of the most likely neuropeptide candidates to be impacted by undernutrition. Indeed, others have shown that chronic undernutrition reduces ARC mRNA for kisspeptin in rodents and sheep (Backholer *et al.* 2010*a*, True *et al.* 2011, Yang *et al.* 2016). While it is common to make inferences on changes in protein from mRNA, our study examined mRNA and protein for ARC kisspeptin, revealing that both are reduced with chronic undernutrition in male sheep. Shorter bouts of nutritional challenges have produced inconsistent results in kisspeptin expression. For example, withholding feed for 3 days in sheep reduced mRNA (Wang *et al.* 2012) and protein (Polkowska *et al.* 2015) for ARC kisspeptin, but feed restriction for 10 days in gilts, which resulted in a significant difference in body weight compared to controls, increased mean LH and LH pulse amplitude while having no effect on mRNA of ARC kisspeptin (Thorson *et al.* 2018). While species differences may exist, another possible explanation for this discrepancy is that the severity of feed restriction (withholding feed vs feed restriction) likely impacts the central expression of kisspeptin. Furthermore, while our model of feed restriction was similar to that of Thorson *et al.* (2018), the length of feed restriction is also likely a contributing factor as several weeks were needed to produce a reduction in LH secretion in our wethers.

Coexpressed within the vast majority of kisspeptin neurons in the ARC (Moore *et al.* 2018), NKB has also been shown to play an active stimulatory role in GnRH/LH pulsatility through direct stimulation of ARC kisspeptin neurons (Goodman *et al.* 2013). While it has been reported that undernutrition reduces mRNA for NKB in rodents (True *et al.* 2011, Yang *et al.* 2016), our study is the first evidence in sheep that undernutrition reduces ARC NKB expression. There is evidence in gilts that feed restriction increases mRNA for ARC NKB (Thorson *et al.* 2018), and while this is not in agreement with the effect of feed restriction on NKB in other species, greater expression of NKB coincided with increased LH concentrations, and further work is needed to determine if NKB plays a role in modulating GnRH/LH pulse amplitude. Furthermore, kisspeptin cell numbers (detected via mRNA and protein) and kisspeptin mRNA abundance per cell were both reduced in our study, but only NKB cell numbers were reduced with no change in NKB mRNA abundance. While there is evidence from non-nutritional models to support differential regulation of peptides within neurons that coexpress kisspeptin and NKB (Cheng *et al.* 2010, Nestor *et al.* 2012, Overgaard *et al.* 2014), one limitation of the current study is that mRNA abundance for NKB was only assessed within cells that expressed both kisspeptin and NKB. Thus, we may have missed any changes that would have occurred in NKB expression within non-kisspeptin neurons, which appear to be more abundant in males than females based on work from others (Goodman *et al.* 2007, Ramaswamy *et al.* 2010, Hrabovszky *et al.* 2012) and our data herein. Albeit, the decrease in total NKB-expressing cells is likely sufficient to result in the loss of stimulatory drive to ARC kisspeptin neurons, and thus reduced GnRH/LH pulsatility.

Dynorphin, also highly coexpressed in ARC neurons with kisspeptin and NKB (Moore *et al.* 2018), differs from these stimulatory neuropeptides in that it has an inhibitory role in regulating GnRH/LH secretion. Dynorphin has been shown to mediate progesterone-negative feedback on LH secretion in adult rats (Gallo *et al.* 1990) and ewes (Foradori *et al.* 2005) and the current working model for dynorphin supports a role in the termination of individual GnRH/LH pulses (Navarro *et al.* 2009, Grachev *et al.* 2012*a*, Weems *et al.* 2018). As for a role linking energy balance to reproduction, food deprivation has inconsistent results with some reporting an increase in dynorphin expression (Berman *et al.* 1997, Herve & Fellmann 1997, Shoham *et al.* 2000), while a more recent report has shown that food deprivation has no effect on ARC dynorphin expression and that chronic food restriction reduces ARC dynorphin expression in ovariectomized female mice (Yang *et al.* 2016). While not a primary focus of this study, we did attempt to measure dynorphin immunoreactivity between FM and FR wethers, but were unable to detect the expression of ARC dynorphin in either group (data not shown). This appears to be a limitation of immunohistochemistry in young animals as we and others (Lopez *et al.* 2016) can detect dynorphin in adult, luteal phase ewes, but not in young male or female sheep. However, given the presence of dynorphin receptors on both GnRH neurons and KNDy neurons (Lopez *et al.* 2016, Weems *et al.* 2016), the nutritional influence of dynorphin on LH secretion could be exerted directly, indirectly, or both, and should receive further investigation.

Although a clear inhibition of undernutrition on kisspeptin and NKB exists in wethers, it is still unclear if this effect of nutrition is a direct regulation or that of afferent input to these essential neurons. Work in sheep using single-cell laser capture has reported that essentially all ARC kisspeptin neurons express leptin receptors (Backholer *et al.* 2010*b*), while another study using immunohistochemistry for pSTAT3, a marker of direct leptin receptor activation, has shown that leptin administration in adult ewes failed to show evidence of functional leptin receptors in ARC kisspeptin neurons (Louis *et al.* 2011). Alternatively, work in sheep has shown that the vast majority of kisspeptin neurons, but not POA kisspeptin or GnRH neurons, express insulin receptors (Cernea *et al.* 2016), thus supporting the idea that insulin may have a direct action on these neurons; however, it remains to be shown whether ARC kisspeptin neurons are activated in response to exogenous insulin during feed restriction. Moreover, while this study examined a central mechanism independent of gonadal sex steroids, further investigation is needed to determine how undernutrition acts within the hypothalamus to increase sensitivity to sex steroid negative feedback on GnRH/LH secretion (Foster & Olster 1985, Beckett *et al.* 1997).

In conclusion, the data here support the hypothesis that chronic feed restriction reduces kisspeptin and NKB expression in the ARC of young, castrated male sheep and that both of these reproductively critical neuropeptides play a role in mediating the effect of undernutrition on GnRH/LH secretion. By using RNAscope and immunohistochemistry, we were able to assess changes in mRNA and protein, respectively, for kisspeptin and NKB within the same study. Furthermore, this is the first evidence in sheep for the nutritional regulation of NKB expression. Taken together, these data clearly demonstrate that even in the absence of gonadal sex steroids, the inhibition of two key stimulatory neuropeptides, kisspeptin and NKB during chronic undernutrition plays an important role in the central suppression of GnRH/LH release.

## Declaration of interest

The authors declare that there is no conflict of interest that could be perceived as prejudicing the impartiality of the research reported.

## Funding

This work was supported by USDA National Institute of Food and Agriculture http://dx.doi.org/10.13039/100005825 Hatch Project 1012905 and by National Institute of Food and Agriculture http://dx.doi.org/10.13039/100005825 Grant no. 2019-67016-29408.

## Author contribution statement

C C N involved in project design and management. A N R, S L S, K S, J R S and C C N contributed to the collection of blood samples, body weight data, and brain tissue. C M M and A N R involved in implementation of IHC. S L S and K H contributed to the development of ovine RNAscope. S L S involved in implementation of RNAscope. C M M and C C N involved in data analysis and writing of this manuscript.
